# Acute superior mesenteric artery occlusion associated with COVID-19 pneumonia: a case report

**DOI:** 10.1186/s40792-022-01360-6

**Published:** 2022-01-10

**Authors:** Masahiro Sukegawa, Satoshi Nishiwada, Taichi Terai, Hiroyuki Kuge, Fumikazu Koyama, Kenji Nakagawa, Minako Nagai, Masayuki Sho

**Affiliations:** Department of Surgery, Nara Medical University, 840 Shijo-cho, Kashihara, Nara 634-8522 Japan

**Keywords:** Superior mesenteric artery occlusion, COVID-19, SARS-CoV-2

## Abstract

**Background:**

The novel 2019 coronavirus disease (COVID-19), which is caused by infection with the severe acute respiratory syndrome coronavirus 2, has spread rapidly around the world and has caused many deaths. COVID-19 involves a systemic hypercoagulable state and arterial/venous thrombosis which induces unfavorable prognosis. Herein, we present a first case in East Asia where an acute superior mesenteric artery (SMA) occlusion associated with COVID-19 pneumonia was successfully treated by surgical intervention.

**Case presentation:**

A 70-year-old man presented to his local physician with a 3-day history of cough and diarrhea. A real-time reverse transcriptase-polymerase chain reaction test showed positive for COVID-19, and he was admitted to the source hospital with the diagnosis of moderate COVID-19 pneumonia. Eight days later, acute onset of severe abdominal pain appeared with worsening respiratory condition. Contrast CT showed that bilateral lower lobe/middle lobe and lingula ground glass opacification with distribution suggestive of COVID-19 pneumonia and right renal infarction. In addition, it demonstrated SMA occlusion with intestinal ischemia suggesting extensive necrosis from the jejunum to the transverse colon. The patient underwent an emergency exploratory laparotomy with implementing institutional COVID-19 precaution guideline. Upon exploration, the intestine from jejunum at 100 cm from Treitz ligament to middle of transverse colon appeared necrotic. Necrotic bowel resection was performed with constructing jejunostomy and transverse colon mucous fistula. We performed second surgery to close the jejunostomy and transverse colon mucous fistula with end-to-end anastomosis on postoperative day 22. The postoperative course was uneventful and he moved to another hospital for rehabilitation to improve activities of daily living (ADLs) on postoperative day 45. As of 6 months after the surgery, his ADLs have completely improved and he has returned to social life without any intravenous nutritional supports.

**Conclusions:**

Intensive treatment including surgical procedures allowed the patient with SMA occlusion in COVID-19 pneumonia to return to social life with completely independent ADLs. Although treatment for COVID-19 involves many challenges, including securing medical resources and controlling the spread of infection, when severe abdominal pain occurs in patients with COVID-19, physicians should consider SMA occlusion and treat promptly for life-saving from this deadly combination.

## Background

The novel 2019 coronavirus disease (COVID-19), which is caused by infection with the severe acute respiratory syndrome coronavirus 2 (SARS-CoV-2), has spread rapidly around the world and has caused many deaths [1]. Although efforts to recognize and manage COVID-19 have been focused on elucidating the respiratory complications, it has now become clear that COVID-19 infection occasionally involves atypical presentations, such as gastrointestinal manifestation and thromboembolic complications [2, 3]. COVID-19 may predispose to both venous and arterial thromboembolism including stroke, myocardial infarction, acute limb ischemia, mesenteric ischemia, deep venous thrombosis, and pulmonary embolism, due to excessive inflammation, hypoxia, immobilization and diffuse intravascular coagulation [2–7].

Acute mesenteric ischemia, especially superior mesenteric artery (SMA) occlusion, is a critical condition with a high mortality rate of 60–80% which requires urgent diagnosis and treatment [8, 9]. COVID-19 and acute SMA occlusion seem to be a very deadly combination knowing the destructive nature of both alone [9]. To date, limited cases of these lethal patient subgroup have been reported in the world. Herein, we report a first case of SMA occlusion in patient with COVID-19 pneumonia successfully treated by surgical intervention in East Asia.

## Case presentation

A 70-year-old man, who had been taking with aspirin, dabigatran and other medication for atrial fibrillation, hypertension, type 2 diabetes mellitus and dyslipidemia, presented to his local physician with a 3-day history of cough and diarrhea. A real-time reverse transcriptase-polymerase chain reaction test of his nasopharyngeal swab specimen showed positive for SARS-CoV-2 nucleic acid, and he was admitted to the source hospital with the diagnosis of moderate COVID-19 pneumonia. He had been administered one liter of supplemental oxygen via nasal cannula and treated by remdesivir and dexamethasone. Antithrombotic therapy with dabigatran and aspirin for comorbidities was continued after his admission.

**Fig. 1:**
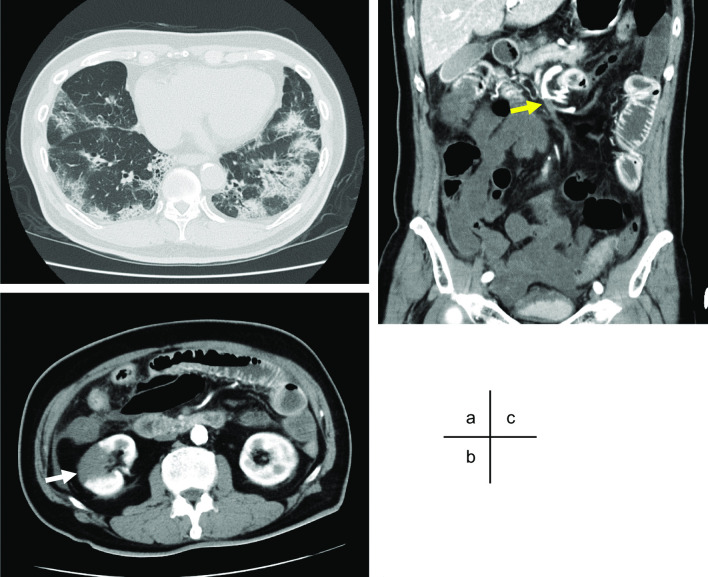
Contrast computed tomography (CT). Contrast CT revealed ground glass opacification with distribution suggestive of COVID-19 pneumonia (**a**), right renal infarction (**b**, white arrow), and superior mesenteric artery occlusion (yellow arrow) with intestinal ischemia suggesting extensive necrosis from the jejunum to the transverse colon (**c**)

Eight days later, acute onset of severe abdominal pain appeared, and he was diagnosed as right renal infarction by contrast computed tomography (CT) at that time. Although heparinization was administered immediately, his clinical course was complicated with worsening abdominal pain and respiratory condition. On the next day, he was transferred to our hospital with severe general condition, and intubated immediately. Contrast CT in our hospital showed that bilateral lower lobe/middle lobe and lingula ground glass opacification with distribution suggestive of COVID-19 pneumonia and right renal infarction. In addition, it demonstrated SMA occlusion with intestinal ischemia suggesting extensive necrosis from the jejunum to the transverse colon (Fig. 1a–c). A retrospective re-review of the CT at the previous hospital showed SMA occlusion was already presented at that time. After consultation with surgery departments, the patient underwent an emergency exploratory laparotomy. During operation, institutional COVID-19 precaution guideline was implemented with appropriate personal protective equipment. Upon exploration, the intestine from jejunum at 100 cm from Treitz ligament to middle of transverse colon appeared clearly necrotic. We resected necrotic bowel and evaluated the blood flow in the preserved intestine by utilizing an indocyanine green fluorescence imaging device, photodynamic eye camera. Although intestinal blood flow was confirmed by the system, considering severe general condition of the patient, we constructed jejunostomy and transverse colon mucous fistula without primary anastomosis.

He was extubated the next day of the surgery and out of intensive care unit on postoperative day 2. The jejunostomy and transverse colon mucous fistula had revealed favorable color with no signs of necrosis. After surgery, the patient was continued on heparinization, and then transitioned to apixaban on postoperative day 11. We performed second surgery to close the jejunostomy and transverse colon mucous fistula with end-to-end anastomosis on postoperative day 22. The postoperative course was uneventful and he required no nutritional treatment despite having only 100 cm small bowel. He moved to another hospital for rehabilitation to improve activities of daily living (ADLs) on postoperative day 45. As of 6 months after the surgery, his ADLs have completely improved and he has returned to social life without any intravenous nutritional supports for short bowel syndrome.

## Discussion

SARS-CoV-2 infection and its disease, COVID-19, have spread rapidly and led the pandemic around the world. COVID-19 has been known a respiratory disease, mainly pneumonia, but also it causes a variety of organ damage [10]. It has been reported worldwide that COVID-19 causes abnormal coagulation from a systemic inflammatory immune response and induces thrombosis-related complications, which is mostly venous thrombosis [5, 6]. Arterial occlusion is less common complication of this disease, but it can involve fatal prognosis [3, 5]. In our literature review on PubMed, total 17 cases (including our case) of SMA occlusion in COVID-19 have been reported from over the world (Table 1) [3, 7, 9–22]. Out of the 14 cases with a description about detailed prognosis in the papers, 5 had died in hospital.

**Table 1:** Literature review: summary of the case reports of SMA thrombosis with COVID-19 infection

Case no.	Authors	Year	Country	Age	Sex	Medical history	Preventive anti-thrombotic therapy	Treatment	Outcomes
1	Amaravathi et al.	2020	India	45	M	None	Missing	Thrombectomy and laparotomy	Unknown
2	Azouz et al.	2020	France	56	M	Missing	Missing	Endovascular thrombectomy/laparotomy	Unknown
3	Bannazadeh et al.	2020	USA	55	M	HT, hyperthyroidism	Heparin	Laparotomy	Alive
4	Barry et al.	2020	France	79	F	None	None	Thrombectomy and laparotomy	Dead
5	Beccara et al.	2020	Italy	52	M	Missing	Heparin	Thrombectomy and laparotomy	Alive
6	Cheung et al.	2020	USA	55	M	HT	Missing	Thrombectomy and laparotomy	Alive
7	Karna et al.	2020	India	61	F	DM, HT	Enoxaparin	Laparotomy	Dead
8	Krothapalli et al.	2020	USA	76	F	Af, CAD, DM, HF, HT	Apixaban	Conservative management	Dead
9	Ucpinar et al.	2020	Turkey	82	F	Af, CKD, HT	Enoxaparin	Conservative management	Dead
10	Vulliamy et al.	2020	UK	75	M	None	None	Thrombectomy and laparotomy	Unknown
11	Balani et al.	2021	India	37	M	None	None	Thrombolysis and thrombectomy	Alive
12	Chaubal et al.	2021	India	9	M	Missing	Missing	Laparotomy	Alive
13	Dinoto et al.	2021	Italy	84	F	CKD, DM, HT	Aspirin	Endovascular thrombectomy	Dead
14	Hanif et al.	2021	Pakistan	20	F	None	None	Laparotomy	Alive
15	Mahrugi et al.	2021	Oman	51	M	Missing	Missing	Thrombectomy and laparotomy	Alive
16	Mitchell et al.	2021	USA	69	M	Missing	None	Thrombectomy and laparotomy	Alive
17	Current case	2021	Japan	71	M	Af, DM, HT	Dabigatran + aspirin	Laparotomy	Alive

*SMA* superior mesenteric artery*, USA* United States of America*, UK* United Kingdom, *Af* atrial fibrillation,* CAD* coronary artery disease*, CKD* chronic kidney disease*, DM* diabetes mellitus*, HF* heart failure, *HT* hypertension

This present case is a first report from East Asia about acute SMA occlusion associated with COVID-19 pneumonia. This is presumably because the distribution of COVID-19 epidemics varies among regions in the world, and the situation of the pandemic revealed relatively stable in East Asia, including Japan, compared to other regions. It suggests that if patients with COVID-19 will increase from the current level, it will be necessary to pay attention to this lethal complication even in areas where the pandemic situation has been currently stable.

Acute mesenteric arterial occlusion most commonly results from thrombosis based on atherosclerotic disease, or embolism where the embolus originates from the left atrium as a consequence of atrial fibrillation [23, 24]. Interestingly, in our literature review, SMA occlusion also occurred in COVID-19 patients who had no risk factors for thromboembolic diseases, which is consistent with previous review [25]. The mechanisms of arterial occlusion in patients with COVID-19 are still unclear. Emerging evidence suggests that COVID-19 is associated with (1) endotheliitis by diffuse endothelial damage and infiltration of inflammatory cells, and (2) a systemic hypercoagulable state caused by hyperinflammation and hypercytokinemia [25–27]. These factors of COVID-19 patients can provide a plausible explanation for the mechanisms of arterial occlusion; highlighting the impact of COVID-19 infection on both thrombosis and embolism. Further fundamental researches to elucidate the mechanism are essential to establish anticoagulation/antiplatelet therapy for preventing this lethal complication.

In our case, due to his medical history of atrial fibrillation, he had been managed with dabigatran since before the thromboembolic complication developed. However, he had multi-site thromboembolism, SMA occlusion and renal infarction. In our literature review, 3 out of 12 patients with a detailed description of their medical history had atrial fibrillation (Table 1). Recently, anticoagulation therapy with heparin and low molecular weight heparin has been reported to be useful for prevention of thrombotic complication with COVID-19 infection [28]. Importantly, despite 7 patients (6 of them had high risk factors for thromboembolic diseases such as atrial fibrillation, diabetes mellitus, hypertension, and dyslipidemia) were maintained with preventive anticoagulation therapy by heparin/low molecular weight heparin or direct oral anticoagulants, they developed SMA occlusion. These results suggest that COVID-19 patients with thromboembolic risk factors are categorized as a high-risk subgroup for SMA occlusion, and stronger anticoagulation, possibly including antiplatelet therapies, may be necessary for those patients, as in our case. In order to establish the preventive strategies for arterial occlusion in COVID-19, further clinical/basic studies using large patient cohorts are required.

From a viewpoint of infection prevention, in patients with COVID-19, even to perform usual clinical examinations and tests, more human and medical resources are required. This severe medical situation may cause exhaustion of medical staffs and inhibit cooperation between infectious disease specialists and abdominal specialists. In this case, it took about 24 h from the onset of abdominal pain to the diagnosis as SMA occlusion. In case that acute onset of severe abdominal pain occurs in patient with COVID-19, physicians should consider the possibility of SMA thrombosis and examine the patient by contrast CT immediately. Furthermore, it is important to collaborate between infectious disease and abdominal specialists to save their lives from this lethal disease.

Necrotic bowel resection is essential for saving lives in most cases of SMA occlusion. In our literature review, 13 cases underwent laparotomy with necrotic bowel resection. It had described the detail of surgical procedures in 9 cased, 4 out of 9 patients were reconstructed with primary anastomosis, and another 5 were constructed intestinal stomas. There were no reports that cases underwent second surgery for intestinal stoma closure. In our case, since the patient's general condition including respiratory function was stable after first surgery, we performed jejuno-transverse colon anastomosis on postoperative day 22. As a result, it was possible to discharge the patient on independent ADLs without intestinal stomas. The decision of whether to perform primary or secondary anastomosis and the optimal timing of secondary anastomosis should be determined, based on the intestinal and general condition, as well as the severity of COVID-19 pneumonia.

## Conclusions

We have reported a first case of SMA occlusion in a patient with COVID-19 pneumonia in East Asia. Intensive treatment including surgical procedures allowed the patient to return to social life with completely independent ADLs. Although treatment for COVID-19 involves many challenges, including securing medical resources and controlling the spread of infection, when severe abdominal pain occurs in patients with COVID-19, physicians should consider SMA occlusion and treat promptly for life-saving from this deadly combination.

## Data Availability

Not applicable.

## Abbreviations

**COVID-19**: Coronavirus disease 19
**SARS-CoV-2**: Severe acute respiratory syndrome coronavirus 2
**CT**: Computed tomography
**SMA**: Superior mesenteric artery
**ADLs**: Activities of daily living
